# Images in infectious diseases: Monkeypox – images of an exhibition

**DOI:** 10.1007/s15010-022-01924-6

**Published:** 2022-10-01

**Authors:** Ana M. Groh, Holger F. Rabenau, Christoph Stephan

**Affiliations:** 1 Center of Internal Medicine, Infectious Diseases Unit, University Hospital Frankfurt, Goethe University, Frankfurt, Germany; 2 Institute for Medical Virology, University Hospital Frankfurt, Goethe University, Theodor-Stern-Kai 7, 60590 Frankfurt, Germany

**Keywords:** Monkeykox, Men having sex with men, HIV, Skin lesions

A 35-year-old man who has sex with men (MSM) approached his HIV treating physician by email, because of new scrotal skin lesions (Fig. 1A), he noticed the day before, appearing with urethral discharge and left-sided, enlarged inguinal lymph node. Self-administered doxycycline, due to suspected syphilis, did not improve symptoms. HIV was detected in December 2019, and antiretroviral therapy was immediately started; medical and sexual transmitted infections (STI)-history was unremarkable.

**Fig. 1:**
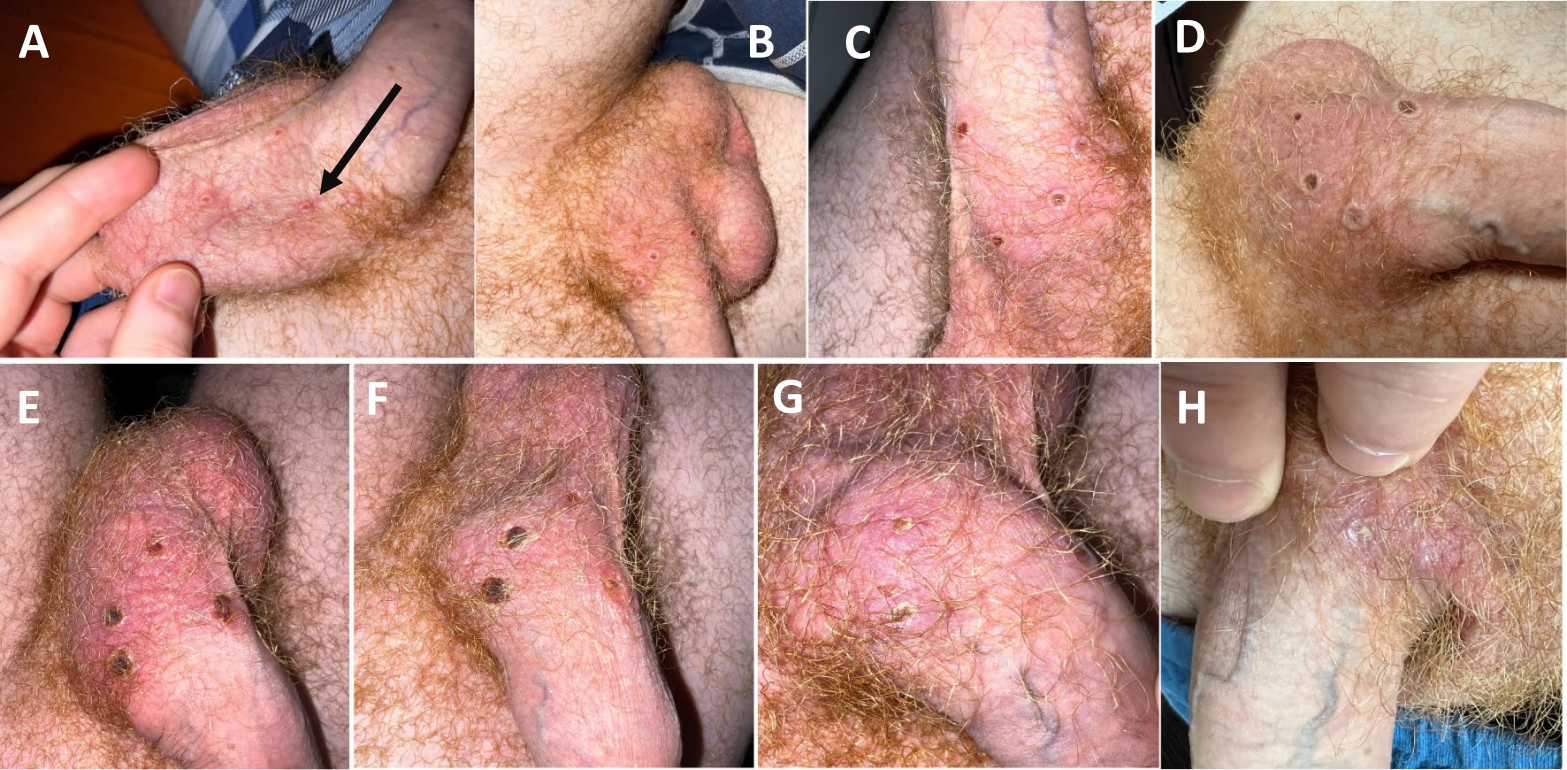
Overtime development of Monkeypox lesions was meticulously documented by the (nurse) patient’s photograph series, after the suspected diagnosis had been mentioned in a first phone call. See detailed diary of skin lesions: **A**—Day 1 (July 5th, 2022): crater-shaped lesions, see arrow: 2 × 2 mm (diameter); **B**—Day 3, hardly changed lesions; **C**—Day 4, the central meltdowns now present enlarged, with more intense redness; **D**—Day 5, lesions start to dry up; **E**—Day 10, central redness enlarge and begin to encrust, lesions’ diameter: now 5 × 5 mm; **F**—Day 15, the lesions are completely covered by reddish crust; **G**—Day 22, scarring regression, crusts start to fall off; **H**—Day 27, scarred healing of lesions

Due to COVID-19 isolation, the first clinical examination was deferred to day 11, revealing four increased circular crater-shaped, scrotal skin elevations with central melting (5 × 5 mm; see Fig. 1E) and no further complaints. Laboratory investigations found slightly elevated C-reactive protein (CRP 1.11 mg/dl, nr < 0.5), normal STI check for syphilis, chlamydia, gonococci and trichomonas, but positive Monkeypox DNA from swap (Ct-value = 18.62; in-house modified *LightMix Modular Monkeypox Virus-PCR*/TibMolBiol, Roche Diagnostics, Mannheim/Germany; Ct-value ≥ 40 = negative). CD4 cell counts (1007/μl, CD4/CD8-ratio 0.79) were normal and HIV-RNA undetectable. On day 29, all skin lesions were dry, scarred and inactive and no new vesicles occurred. Therefore, health authority-imposed Monkeypox isolation was finally lifted and patient returned to work as elderly care nurse.

Nucleic acid assays and electron microscopy (see Fig. 2) may support clinical Monkeypox diagnosis in MSM context [1]. Best supportive care of lesions will be most frequently treatment for immunocompetent individuals, as specific antiviral therapy is unavailable [2]. Facing case numbers in Germany [3], a high level of suspicion for Monkeypox visual diagnosis is warranted and this presentation of overtime skin lesions may help for individual timing assignment.

**Fig. 2:**
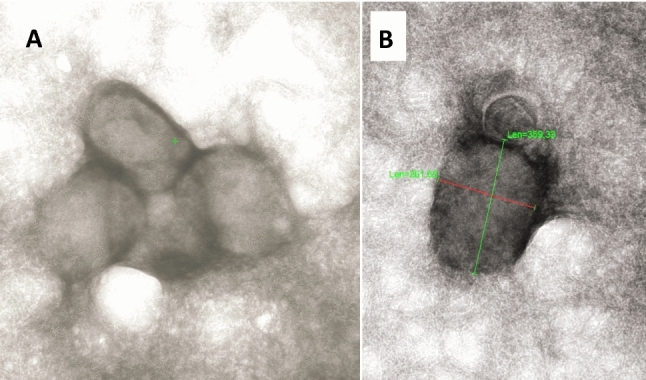
Monkeypox virus: Scanning electron microscopy (sample obtained from another individual in Frankfurt). Panel **A** shows a group of 4 typically box-shaped (Monkey-) Pox viruses, panel **B** shows measurement procedure outcome (longitudinal diameter = 359 nm)

## Data Availability

The authors confirm that the data supporting the findings of this study were raised from clinical routine in Frankfurt University Hospital outpatient department and are available within this article.
